# Combinatorial Extracellular Matrix Microenvironments for Probing Endothelial Differentiation of Human Pluripotent Stem Cells

**DOI:** 10.1038/s41598-017-06986-3

**Published:** 2017-07-26

**Authors:** Luqia Hou, Joseph J. Kim, Maureen Wanjare, Bhagat Patlolla, John Coller, Vanita Natu, Trevor J. Hastie, Ngan F. Huang

**Affiliations:** 1 Stanford Cardiovascular Institute, Stanford University, Stanford, CA USA; 2 Veterans Affairs Palo Alto Health Care System, Palo Alto, CA USA; 3 Stanford Functional Genomics Facility, Stanford, CA USA; 4 Department of Statistics, Stanford University, Stanford, CA USA; 5 Department of Biomedical Data Science, Stanford, CA USA; 6 Department of Cardiothoracic Surgery, Stanford, CA USA

## Abstract

Endothelial cells derived from human pluripotent stem cells are a promising cell type for enhancing angiogenesis in ischemic cardiovascular tissues. However, our understanding of microenvironmental factors that modulate the process of endothelial differentiation is limited. We examined the role of combinatorial extracellular matrix (ECM) proteins on endothelial differentiation systematically using an arrayed microscale platform. Human pluripotent stem cells were differentiated on the arrayed ECM microenvironments for 5 days. Combinatorial ECMs composed of collagen IV + heparan sulfate + laminin (CHL) or collagen IV + gelatin + heparan sulfate (CGH) demonstrated significantly higher expression of CD31, compared to single-factor ECMs. These results were corroborated by fluorescence activated cell sorting showing a 48% yield of CD31^+^/VE-cadherin^+^ cells on CHL, compared to 27% on matrigel. To elucidate the signaling mechanism, a gene expression time course revealed that VE-cadherin and FLK1 were upregulated in a dynamically similar manner as integrin subunit β3 (>50 fold). To demonstrate the functional importance of integrin β3 in promoting endothelial differentiation, the addition of neutralization antibody inhibited endothelial differentiation on CHL-modified dishes by >50%. These data suggest that optimal combinatorial ECMs enhance endothelial differentiation, compared to many single-factor ECMs, in part through an integrin β3-mediated pathway.

## Introduction

Human endothelial cells (ECs) derived from pluripotent stem cells are a potential therapeutic cell type for the treatment of ischemic cardiovascular diseases such as peripheral arterial disease and myocardial infarction^1–8^. Unlike bone marrow-derived endothelial progenitor cells that have a limited expansion potential, ECs can be harvested indefinitely from pluripotent stem cells, owing to their infinite expansion potential. Our knowledge of the microenvironmental factors and underlying fundamental biology that regulate endothelial differentiation is lacking and is a critical bottleneck to the efficient derivation and clinical translation of pluripotent stem cell-derived ECs.

To address this critical gap in knowledge, we studied the role of extracellular matrix (ECM) proteins present in the basement membrane of the endothelium, which are well-recognized to impart dynamic signaling to ECs^9^. The basement membrane ECM consists of a milieu of proteins including collagen IV, fibronectin, laminin, and heparan sulfate proteoglycans^10^. ECMs such as collagen IV and fibronectin have been reported to enhance endothelial differentiation^11, 12^. However, a limitation of such single-component ECMs is that it simplifies the multi-component ECMs that comprise the endothelial basement membrane. Given the importance of the multi-component ECMs in modulating EC function and phenotype, we sought to examine the role of combinatorial ECMs on endothelial differentiation systematically using an arrayed microscale platform.

High-throughput techniques have been employed to develop biomaterials arrays for tissue engineering and drug delivery approaches, as well as for improving and understanding stem cell differentiation and fate commitment. The ECM microarray platform provides a high-throughput and systematic way to probe mechanisms of stem cell behavior and function. Flaim *et al*. developed a five-component ECM microarray to study the effect of 32 different combinatorial ECMs on the maintenance of primary rat hepatocyte and differentiation of mouse ESCs toward hepatic fate^13^. Later, Brafman *et al*. used this ECM array approach and studied the effects of combinatorial ECMs on modulating hepatic stellate cell phenotype^14^. In addition, the microarray platform was used to select synthetic polymers that improve attachment, proliferation and self-renewal among multiple human ESCs lines over five passages^15^. More recently, it was reported that ECM microarrays were used to study the ECMs that regulate endodermal differentiation in human ESCs^16^, as well as identify synthetic polymers that promote neuronal differentiation of progenitor cells^17^.

Accordingly, we hypothesized that combinatorial ECMs enhance endothelial differentiation from pluripotent stem cells, when compared to single-factor ECMs. Using a microscale high-throughput platform for simultaneous screening of endothelial differentiation in 63 unique combinatorial ECM microenvironments, we demonstrate that multi-component ECMs such as collagen IV + heparan sulfate + laminin (CHL) or collagen IV + gelatin + heparan sulfate (CGH) show significant enhancement over single-factor ECMs in endothelial differentiation, in part by integrin-specific signaling. This approach enables full-factorial analysis of interaction effects between ECMs, which may be critical for understanding how stem cells differentiate in response to complex ECM cues.

## Results

### Combinatorial ECMs modulate endothelial differentiation in iPSCs and ESCs

**Figure 1:**
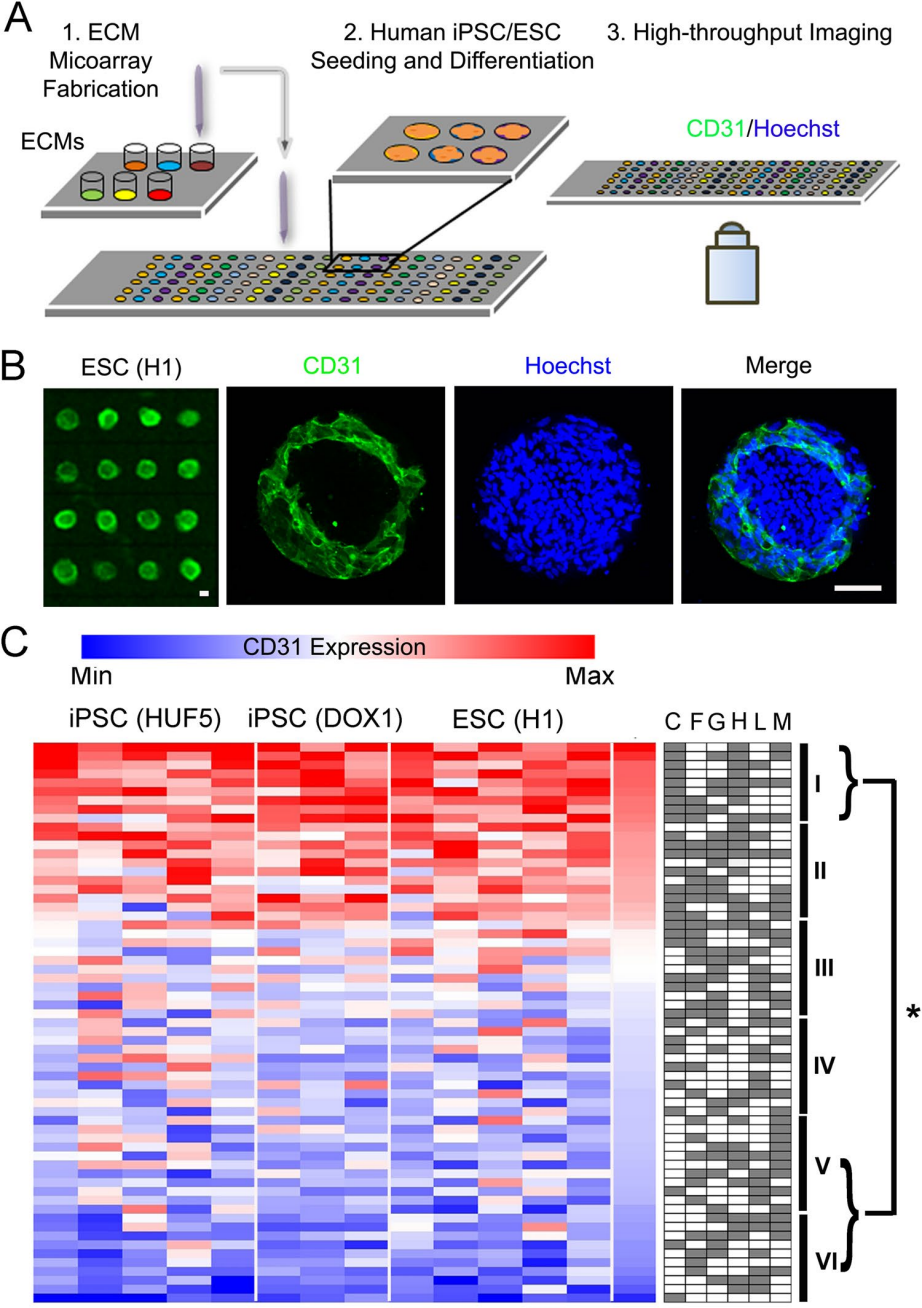
Multi-component ECMs promote endothelia differentiation in human iPSCs and ESCs. (**A**) Schematic diagram of ECM microarray fabrication and endothelial differentiation. First, the ECM microarray was fabricated from six ECMs and 63 distinct combinatorial ECMs. Human iPSCs or ESCs were then seeded and differentiated on the microarray. Afterwards, high-throughput imaging was performed using the ImageXpress Micro high-content imaging system. (**B**) ImageXpress (left) and confocal (right) images of representative ESCs on ECM microenvironments after 5 days of endothelial differentiation. The confocal images shown at high magnification depict endothelial differentiation on an island of matrigel. Green, CD31; Blue, Hoechst 33342. Scale bar, 100 µm. (**C**) Heat map of normalized CD31 expression for each independent microarray slide generated from three cell lines: iPSCs (HUF5, n = 5), iPSCs (DOX1, n = 3) and ESCs (H1, n = 5). Integrated fluorescence data was measured and normalized to cell numbers from each ECM microenvironment. The normalized average data was log_2_-transformed and globally normalized to the mean of all 63 ECM combinations to generate the Z scores, which were ranked in the heat map of CD31 expression from low (blue) to high (red) levels. Each column represents an independent ECM microarray slide, whose data was averaged among the 6 replicates per slide. The identity of all 63 combinatorial ECMs is shown on the right in which white or grey boxes denotes the absence or present, respectively, of an ECM component. Combinatorial ECMs were grouped into six tiers from high (Tier I) to low (Tier VI). Tier I combinatorial ECMs significantly promoted CD31 expression when compared to any ECMs in Tier V and VI. *Denotes P < 0.05.

Combinatorial ECM microarrays consisting of ECMs found in the endothelial basement membrane were fabricated and characterized as previously described (Fig. 1A)^15, 18^. Each ECM microarray slide consisted of 63 unique ECM compositions derived from the 6 ECM proteins at equal mass ratios (1:1, 1:1:1, etc.). Each of the 63 ECM compositions were printed onto the microarray in replicates of 6, resulting in a total of 378 ECM individual islands that were 0.3 mm in diameter. The identity of all 63 combinatorial ECMs is shown on the right in which white or grey boxes denotes the absence or present, respectively, of an ECM component. Human iPSCs (HUF5 and DOX1) and ESCs (H1) attached reproducibly among replicates of the same combinatorial ECM. Consistent cell attachment among replicates of the same ECM composition can be observed in Fig. 1B. To assess endothelial differentiation efficiency across three cell lines, we quantitatively compared the expression of CD31, which is a characteristic phenotypic marker of ECs. We used well-established image analysis software and Z-score standardization algorithms^16, 19^ to compare the intensity of CD31 among combinatorial ECMs after normalization by cell number. The results were then displayed in a heat map that ranked the combinatorial ECMs from highest to lowest normalized CD31 expression (Fig. 1C). For greater ease in referring to the ECM microenvironments, the ECMs were grouped into six tiers. The ECMs that resulted in the highest CD31 expression were located in Tier I, and they consisted predominantly of combinatorial ECMs such as C + M + L (CML), C + H + L (CHL), and C + H (CH). Among single-factor ECMs, H and G were ranked within Tier II, whereas L was ranked in Tier IV, M was ranked in Tier V, and F and C were ranked in the bottom-most tier. All ECMs in Tier I enhanced endothelial differentiation significantly comparing to each of the ECMs in Tiers V and VI (P < 0.05, Supplementary Figure I). These results strongly indicated that combinatorial ECMs in Tier I promoted significantly higher expression of CD31 than many single-factor ECMs, and this finding was consistent across all 3 cell lines.

**Figure 2:**
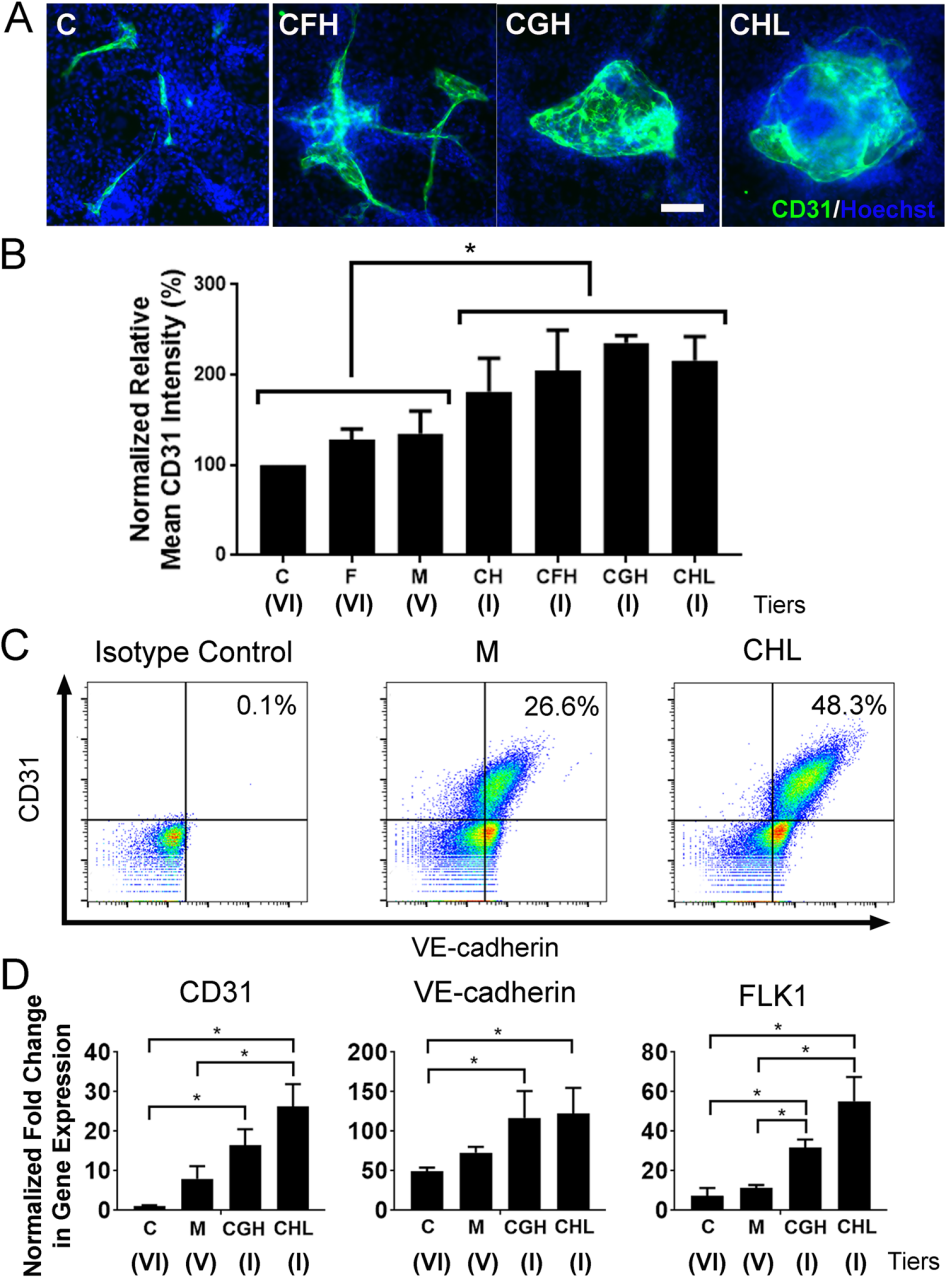
Conventional cell culture platform validates the effect of combinatorial ECMs on endothelial differentiation. (**A**) Representative confocal images of differentiated ESCs (H1) after 5 days of differentiation. Endothelial differentiation based on CD31 expression was assessed on chamber slides pre-coated with representative combinatorial or single-factor ECMs: CHL (Tier I); CGH (Tier I); CFH (Tier I); CH (Tier I); M (Tier V); F (Tier VI) and C (Tier VI). Green: CD31, Blue: Hoechst 33342. There was a significant increase in the amount of CD31 expression in Tier I multi-component ECMs such as CHL and CGH, compared to single-factor ECMs like F and C in Tier VI. (**B**) Quantification of normalized CD31 protein intensity shows a significant increase in CD31 expression between single-component ECMs from Tier VI and multi-component ECMs in Tier I (n ≥ 6). (**C**) Characterization of ESC (H1) differentiation efficiency by FACS. The CD31^+^/VE-cadherin^+^ cell population on Tier I CHL-coated plates (48.3%) was higher than that on Tier V M-coated plates (26.6%). (**D**) Quantitative PCR shows significant increase in gene expression of CD31, VE-cadherin and FLK1 when ESCs underwent endothelial differentiation on Tier I ECM combinations CHL and CGH (n = 3). *Denotes P < 0.05.

To validate these results from an ECM microarray in a conventional cell culture format, the ESCs were differentiated on ECM-coated chamber slides to compare the normalized expression of CD31 among high scoring combinatorial ECMs to representative single-factor ECMs: CHL (Tier I), CGH (Tier I), CFH (Tier I), CH (Tier I), M (Tier V), F (Tier VI), and C (Tier VI) (Fig. 2A,B). After 5 days of differentiation, the normalized relative expression levels followed a similar expression pattern to that on the ECM microarray: CHL (235.1 ± 8.2%), CGH (215.6 ± 26.82%), CFH (205 ± 44.60%), CH (181.1 ± 37.3%), M (134.9 ± 25.0%), F (127.8 ± 12.1%), and C (100 ± 0%). These results demonstrated a significant relative increase in CD31 expression in Tier I combinatorial ECM, CHL, when compared to single-factor ECMs C or F (P < 0.05). In addition, fluorescence-activated cell sorting (FACS) was performed to quantify the cell population of CD31^+^/VE-cadherin^+^ ESC-derived ECs. As shown in Fig. 2C, the CD31^+^/VE-cadherin^+^ cell population was 48.3% in the CHL-coated plates, compared to 26.6% on M-coated plates, which concurs with the protein expression data (Fig. 2B). Besides protein expression, gene expression analysis of endothelial markers (CD31, VE-cadherin and FLK1) also showed a significant enhancement when the cells were differentiated on ECMs in Tier I, such as CHL and CGH (Fig. 2D). We further showed that the CD31^+^ cells on CHL-coated dishes co-expressed VE-cadherin and CD105 (endoglin), and could take up acetylated low density lipoprotein as phenotypic and functional markers of CD31^+^ ESC-derived ECs (Supplementary Figure II). Together, these results concurred with findings from the microarray analysis of CD31 expression, suggesting that combinatorial ECMs in the Tier I could augment endothelial differentiation, when compared to single-factor ECMs from lower tiers.

### Analysis of multi-factorial interactions and non-intuitive cellular response

**Figure 3:**
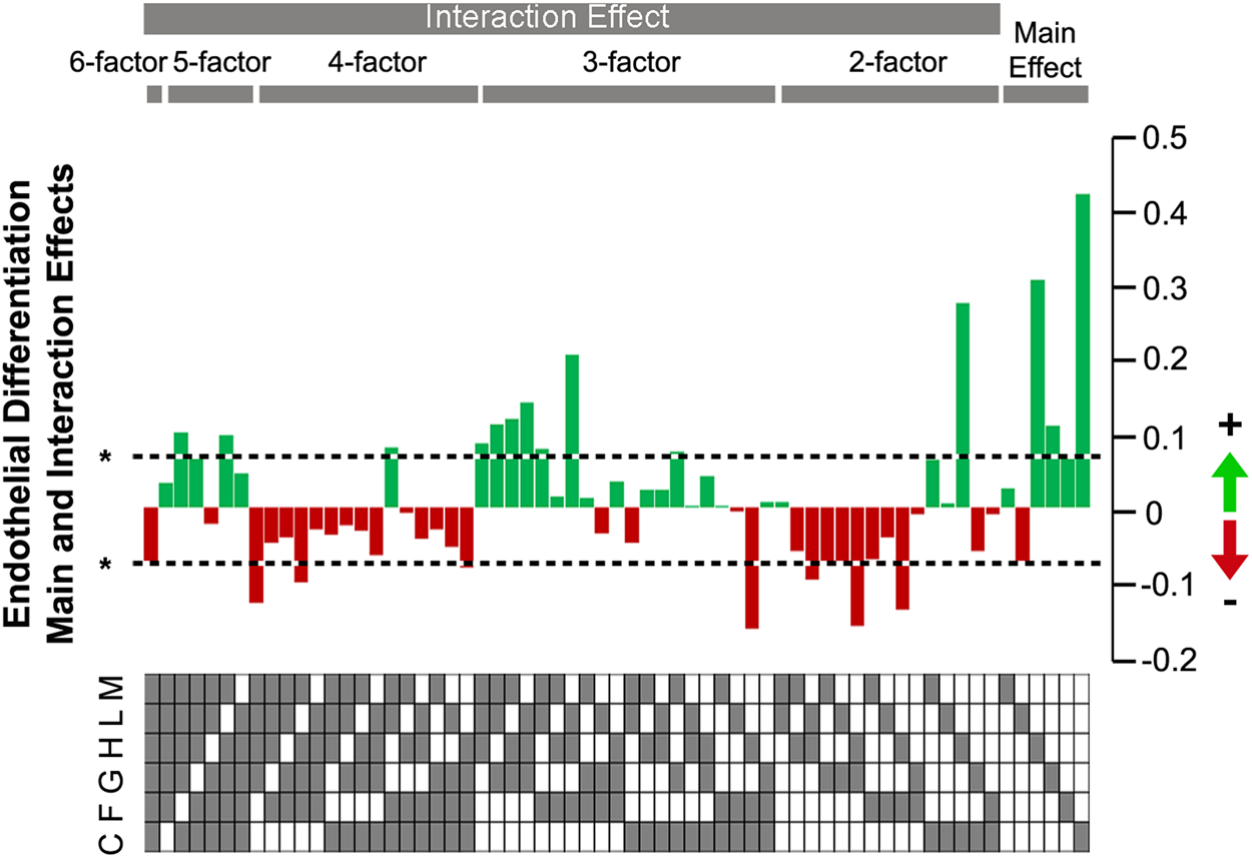
Main and multi-factorial ECM interactions based on CD31 expression. Based on the CD31 expression data generated from iPSCs (HUF5, n = 5), iPSCs (DOX1, n = 3) and ESCs (H1, n = 5), the ECM combinations were grouped and ranked by 1-factor, 2-factor, 3-, 4-, 5- and 6-factor combinations. Main and multi-factorial ECM effects were analyzed and compared as coefficients in a multi-way ANOVA model. The graph depicts the main effects measured by the change in CD31 expression when the listed ECM component is present vs. absent. A positive effect is indicated by green bars, whereas red bars denote negative effects. 0 represents neither positive nor negative effect. The dotted line indicates *P < 0.05.

Besides examining the effects of combinatorial ECMs on endothelial differentiation, we further applied this combinatorial approach to assess the main and multi-factorial ECM effects. This analysis provides fundamental insights into how complex ECM microenvironments influence endothelial differentiation. Based on full-factorial analysis, C, G, and H had significant positive effects in augmenting CD31 expression, whereas L had negative effects and reduced CD31 expression (P < 0.05, Fig. 3). Besides main effects, our data also suggest many significant multi-factorial interactions up to the highest order that also impact CD31 expression.

**Figure 4:**
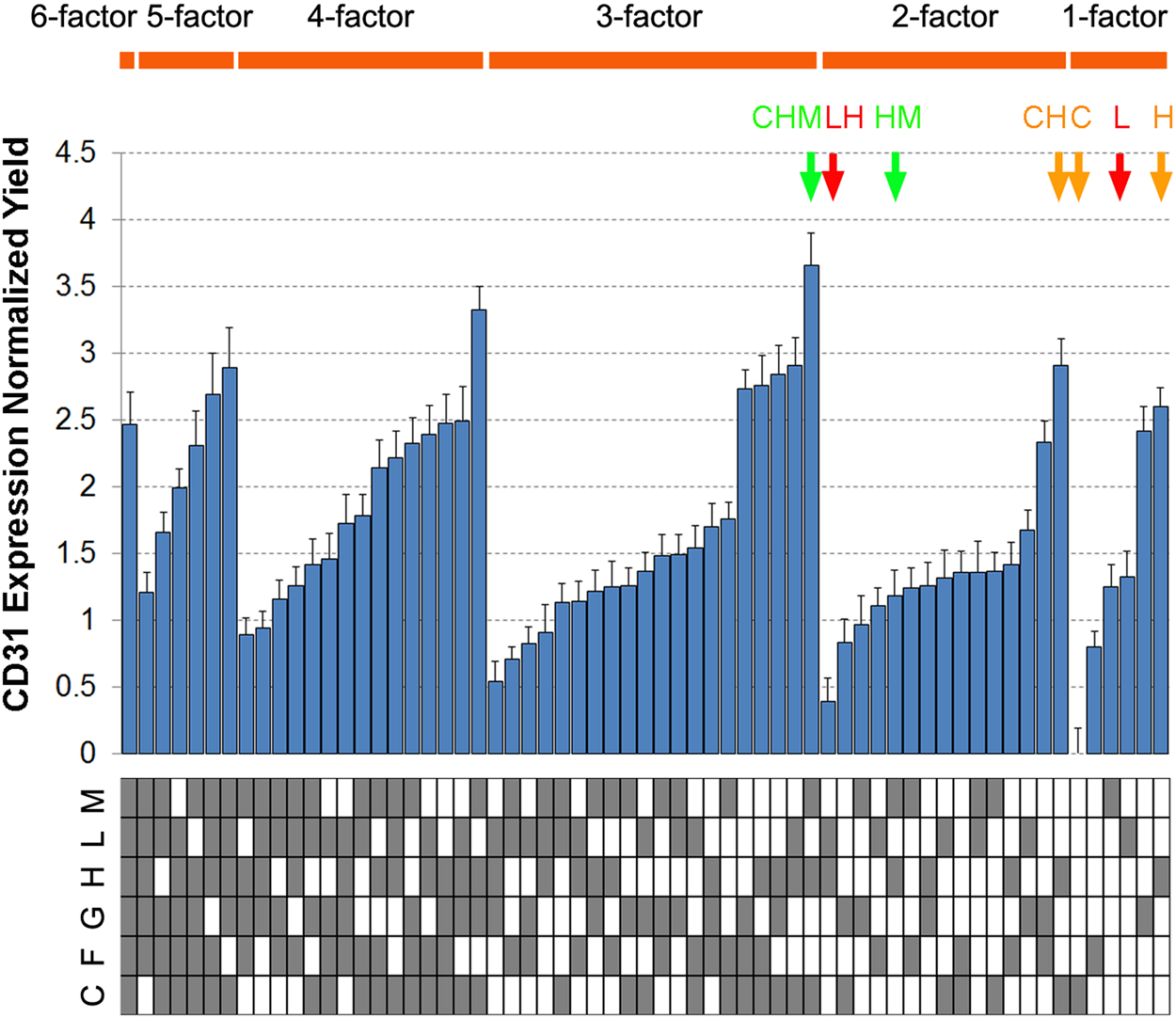
Non-additive interaction effects among ECM combinations on endothelial differentiation. Based on the CD31 expression data generated from iPSCs (HUF5, n = 5), iPSCs (DOX1, n = 3) and ESCs (H1, n = 5), examples of synergistic, inhibitory and redundant interactions were labeled in green, red, and orange respectively. Green: C + H + M (3.66 ± 0.25) indicates a synergistic effect as C (0 ± 0.19) and H + M (1.18 ± 0.19) had a lower CD31 expression score, compared to the three combined. Red: L (1.33 ± 0.19) and H (2.60 ± 0.14) showed inhibitory effect as L + H (0.39 ± 0.18) resulted in much lower CD31 expression than each individual ECM. Orange: C (0 ± 0.19) and H (2.60 ± 0.14) showed a redundant effect as CD31 expression on C + H (2.91 ± 0.20) was similar to that on H alone.

Based on ESC fate determination response to combinatorial ECMs, non-intuitive interaction effects among ECM components could be systematically observed. For example, in comparing the CD31 expression among ECM combinations (Fig. 4), single-factor C or two-factor H + M (HM) resulted in CD31 expression at levels of 0 ± 0.19 and 1.18 ± 0.19, respectively. However, when C and HM were combined together, the three-factor combination of C + H + M produced a synergistic increase in the normalized yield of 3.66 ± 0.25 (in green). In addition, some interactions identified were redundant in nature. For example, in comparison to single-factor H (2.60 ± 0.15), the addition of single-factor C (0 ± 0.19) showed no marked change to normalized CD31 expression yield in the two-factor combination of C + H (2.91 ± 0.20, in orange), suggesting that H played a dominant role over C in modulating endothelial differentiation. In another example, single-factor L modulated CD31 expression at normalized yields of 1.33 ± 0.19. However, the two-factor combination L + H reduced the normalized yields to 0.39 ± 0.18 (in red), suggesting an inhibitory interaction of the two individual ECMs. These examples highlight the power of this combinatorial ECM microarray platform to provide new insights into endothelial differentiation in response to complex ECM environments, which would otherwise be difficult to achieve in conventional tissue culture plate formats.

### Integrin expression during endothelial differentiation

**Figure 5:**
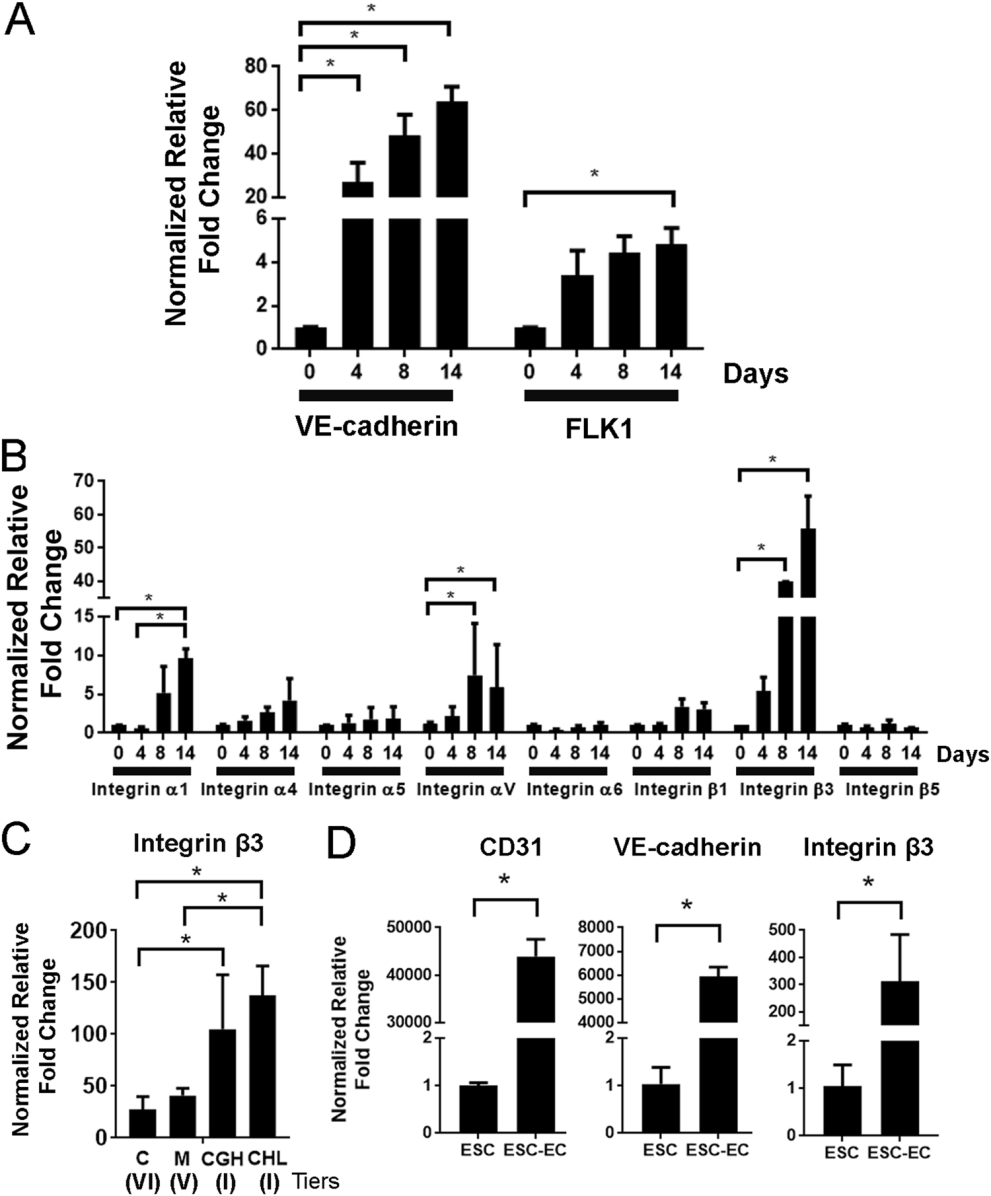
Multiple integrin subtypes were upregulated during endothelial differentiation in ESCs. (**A**) Using a related 14-day endothelial differentiation protocol that encourages endogenous ECM production, gene expression analysis showed that the endothelial markers VE-cadherin and FLK1 were upregulated during the course of differentiation. (**B**) Concomitant with upregulation of endothelial genes, integrins α1, αV and β3 were also significantly upregulated through 14 day endothelial differentiation. (**C**) Using a 5-day differentiation protocol, non-purified ESCs (H1) on Tier I combinatorial ECMs (CHL and CGH) had a significant increase in integrin β3 gene expression, compared to single-factor ECMs such as C (Tier VI) and M (Tier V) (n = 3). (**D**) Purified ESC-derived endothelial cells (ESC-ECs) showed a significant increase in CD31, VE-cadherin, and integrin β3 gene expression, compared to ESCs (H1) (n = 4). *Denotes P < 0.05.

Since ECMs modulate cell behavior by binding to integrin transmembrane receptors, we first quantitatively assessed the temporal gene expression pattern of integrin subunits, compared to that of endothelial phenotypic markers. Using a related differentiation protocol that relies on endogenously produced ECMs during embryoid body formation, the gene expression time course of endothelial phenotypic markers such as VE-cadherin and FLK1 showed a progressive increase in expression over the course of differentiation in ESCs (Fig. 5A). Concomitantly, integrin subunits α1, α_V_ and β3 were also significantly upregulated by 10, 5 and 50 folds, respectively, in comparison to day 0 (Fig. 5B) (P < 0.05). Similarly, iPSCs (DOX1) showed an upregulation of these integrin subunits over the course of endothelial differentiation (Supplementary Figure III). These results suggest that integrin β3 was associated with endothelial differentiation in ESCs and iPSCs. To confirm that integrin β3 directly modulated the endothelial differentiation in the presence of combinatorial ECMs, we examined the gene expression of integrin β3 in ESCs differentiated on combinatorial ECMs using our 5-day differentiation protocol. Relative to cells at the start of differentiation, integrin β3 was upregulated by 137.6 ± 28.24 and 105 ± 52.2 fold in Tier I ECMs such as CHL and CGH, in comparison to single factor components C (27.1 ± 12.7) and M (41 ± 6.9) (Fig. 5C). Furthermore, when ESC-derived ECs were purified by flow activated cell sorting for CD31^+^/VE-cadherin^+^ expression after 5 days of differentiation, these cells showed a >300-fold increase in integrin β3 expression, concomitant with an increase in CD31 and VE-cadherin, compared to undifferentiated ESCs (Fig. 5D). These studies suggest that integrin β3 expression may be an important mechanotransducer of ECM cues that promote endothelial differentiation.

### Abrogation of endothelial differentiation by inhibition of integrin β3

**Figure 6:**
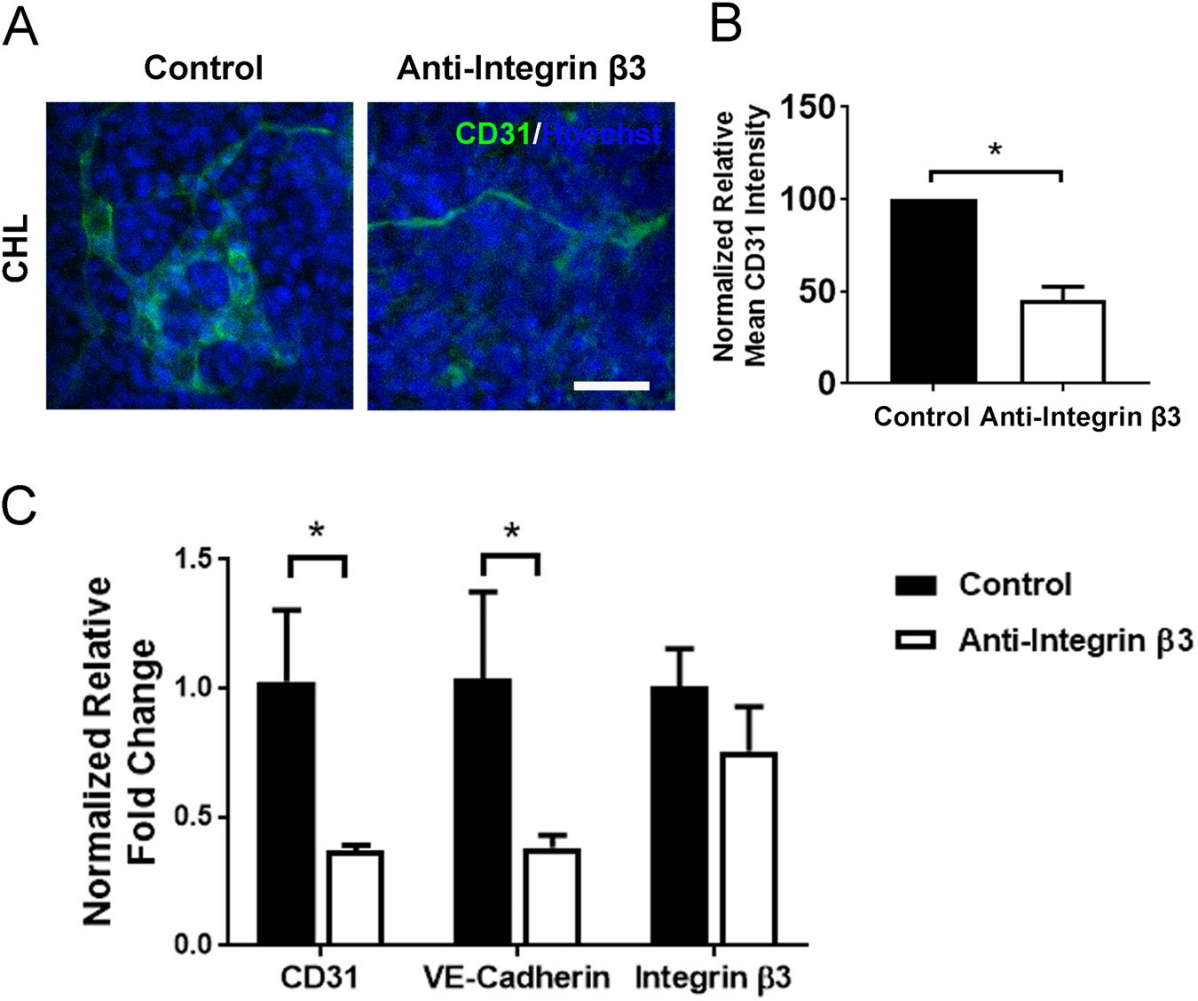
Inhibition of integrin β3 using neutralization antibody. (**A**) Representative confocal images of ESCs (H1) differentiated for 5 days on Tier I combinatorial ECM (CHL). Prior to the start of differentiation and throughout the differentiation time course, ESCs were treated with integrin β3 neutralizing antibody (right) or IgG antibody (left). (**B**) Quantification of CD31 protein expression shows a significant reduction in endothelial differentiation as a result of integrin β3 inactivation (n = 3). (**C**) Quantitative PCR shows a significant reduction in gene expression in CD31 and VE-cadherin (n = 3). *Denotes P < 0.05.

In order to demonstrate the importance of integrin β3 in modulating endothelial differentiation of top tier ECMs, ESCs in suspension were incubated with integrin β3 neutralization antibody before seeding onto cell culture chambers coated with CHL (Tier I). The neutralization antibody was supplemented in the media for the entire duration of endothelial differentiation. As shown in Fig. 6A,B, the cells treated with integrin β3 antibody exhibited reduced normalized CD31 protein expression of 45.4 ± 7.2%, relative to cells treated with isotype control antibodies (P < 0.05). Meanwhile, cell numbers were not affected by the addition of neutralization antibodies (data not shown). Similar experiments were performed using ESCs (H1) on matrigel-coated tissue culture chamber slides. When cells were treated with antibody against integrin β3 or α1, endothelial differentiation was downregulated significantly, compared to cells treated with isotype control antibody (Supplementary Figure IV). Concomitant to protein expression changes, the presence of integrin β3 neutralization antibody abrogated the expression of endothelial genes VE-cadherin and CD31 (Fig. 6C). These results demonstrated that endothelial differentiation from combinatorial ECMs such as CHL is regulated in part by integrin β3-mediated signaling.

## Discussion

In this work, we successfully developed a high-throughput ECM microarray to evaluate the role of combinatorial ECMs in stem cell differentiation. The main findings of this study are: (1) the Tier I combinatorial ECMs (ie CHL and CGH) were capable of promoting endothelial differentiation by increasing the expression of CD31 at the protein and transcriptional level (Figs 1 and 2), when compared to other ECM compositions such as C or F in Tiers V and VI, respectively; (2) the ECM microarray results could be validated at the macroscopic level in conventional cell culture formats (Fig. 2); (3) main and multi-factorial interactions analysis provided insight into the effect of ECMs on stem cell endothelial differentiation (Fig. 3); (4) the ECM microarray platform identified non-intuitive cellular responses (synergistic, redundant or inhibitory effect) that shed light into how cells respond to complex ECM cues (Fig. 4); and (5) integrin β3 activation was associated with the enhancement of CD31 expression, as neutralization of integrin β3 activity abrogated the positive effect of Tier I ECM combinations on endothelial differentiation (Figs 5 and 6). Based on these findings, endothelial differentiation of human pluripotent stem cells could be augmented by the use of combinatorial ECMs in Tier I, in part through integrin β3 triggered signaling pathway. To the best of our knowledge, this is the first report of endothelial differentiation among human ESC and iPSC lines using the high-throughput ECM microarray platform.

The rationale for investigating the role of ECMs is guided by our understanding of how ECMs regulate vascular formation and survival during embryonic development. During the early stage of mesoderm development of the blastocyst, fibronectin and laminin are expressed and play an important role in cell migration^20^. Integrins α5β1 and α6β1, which are the main receptors for fibronectin and laminin, respectively^21, 22^, regulate embryonic lethality. Mutation in integrin α5 has been shown to cause defects by day 9 of gestation, leading to embryonic lethality around days 10–11 in mice^23^. In addition, Liu *et al*. showed that insoluble ligands for both α5β1 and α6β1 integrins support mesoderm differentiation^24^. Furthermore, integrins αvβ3 and αvβ5 are known to bind to fibronectin, vitronectin, and osteopontin and regulate vascular development^25, 26^. Integrins such as αvβ3 have been shown important in blood vessel maturation, as microinjection of anti-αvβ3 monoclonal antibody disrupts the normal pattern of vascular development in quail embryos^27^. In particular, integrin β3 could serve as an important receptor that interacts with the RGD binding domain of collagen IV or laminin^28^, which were among the Tier I ECM combinations. Together, these data suggest that ECMs and integrins play a critical role in embryonic development and provide strong evidence that endothelial differentiation could be regulated through cell-ECM interactions.

Although the ECM microarray used in this study only has 400 ECM islands, this microarray platform has the capacity of testing thousands of proteins in microliter volumes at the same time in a high-throughput manner. Compared to traditional cell culture dishes, this platform only requires a small number of cells (2 × 10^6^) and little media (5 mL), which is cost-effective and less time consuming. More importantly, with advanced statistical and informatics analysis, the microarray data provides the assessment of multi-factorial interaction effects between ECMs in a systematic and quantitative way. For example, our data suggest that when multiple ECM components are present, synergistic, redundant and inhibitory effects were observed in endothelial differentiation of pluripotent stem cells (Figs 3 and 4). This multi-factorial interaction was significant through up to 6-factor interactions. This indicates that ECM interactions are important in guiding endothelial differentiation.

The ECMs used in the microarray were selected based on their abundance in the basement membrane of blood vessels under physiological conditions, or due to their common usage for endothelial differentiation of pluripotent stem cells on tissue culture Petri dishes. Specifically, collagen IV, laminin and heparan sulfate are abundant ECMs in the basement membrane of blood vessels^10^, whereas during angiogenesis fibronectin is secreted by ECs into the provisional matrix^29, 30^. Furthermore, gelatin^31^ and matrigel^32^ are commonly used ECMs during endothelial differentiation either using embryonic body formation approach or feeder free approach. Although this study is limited by assessing only these six ECMs, it sets an important stage for future studies that have an increase in the number and complexity of the ECM components.

The signaling pathways in which combinatorial ECMs modulate stem cell fate commitment or lead to non-intuitive interactions is still largely unknown. Based on previous studies, the possible mechanism may include factors of physical properties such as rigidity, protein composition of matrices, and/or the available binding sites for integrin heterodimers. Furthermore, the actin cytoskeleton, as well as a number of transcription factors and chromatin remodeling enzymes, was reported as important components of the mechanosignaling cascade. In addition, RhoA and its downstream effector, Rho kinase, has shown critical role in cell differentiation^33^. More importantly, the synergistic and inhibition effects could result from the crosstalk between integrin and growth factor signaling among different matrix molecules^34^.

To date there are many reports of endothelial differentiation protocols for pluripotent stem cells, most of which rely on signaling from soluble factors and/or single-factor ECMs for differentiation. For example, Blancas *et al*. developed a chemically defined protocol that involves the addition growth factors (ie VEGF-A, BMP-4, and basic fibroblast growth factor (bFGF)) and ECMs, in which fibronectin was shown to be more potent than other ECMs in inducing endothelial differentiation^35^. Lian *et al*. reported that they utilized an endothelial differentiation protocol based on WNT signaling induction using GSK3 inhibitor CHIR99021 and matrigel^36^. More than 50% CD34^+^/CD31^+^ endothelial progenitors were obtained in five days. However, further expansion up to two months was required to obtain mature CD31^+^/VE-cadherin^+^/CD34^−^ pluripotent stem cell-derived ECs. More recently, Glaser *et al*. further optimized the previous protocol by defining the differentiation into two stages, first from pluripotent stem cell to FLK1^+^ vascular progenitor cells (VPCs), and then from VPCs to VE-cadherin^+^ pluripotent stem cell-derived ECs^37^. The cell seeding density, matrix substrate (fibronectin, collagen IV, and gelatin) and growth factor (VEGF-A and bFGF) concentrations were studied in four different cell lines. In addition, Palpant *et al*. developed a common platform to generate pluripotent stem cell-derived ECs from hemogenic mesoderm using growth factors, small molecules, and matrigel, and they reported >90% purity^38^.

In comparison to such differentiation protocols that yield high purity, the role of combinatorial ECMs on differentiation may be easily masked by the potency of the soluble factors. In this work we intentionally selected a differentiation protocol that employs only a small number of growth factors (namely VEGF-A and BMP4), and has previously shown only to give rise to 10–20% endothelial differentiation efficiency^39^. Along with reducing the differentiation period of 5 days, we believe our differentiation protocol creates a window in which the effect of combinatorial ECMs on endothelial differentiation can be measurable. However, our findings are limited by the use of serum in the differentiation media, which is subjected to the batch-to-batch variability^35^.

In conclusion, we developed a high-throughput ECM microarray to systematically study combinatorial ECMs in regulating endothelial differentiation, and identified that combinatorial ECMs such as CHL and CGH were able to significantly upregulate differentiation efficiency, compared to single-factor ECMs, through an integrin β3 activated signaling pathway.

## Methods

### Fabrication of ECM microarray slides

The ECM microarray was fabricated from single-component ECMs or multi-component mixtures of the following ECM proteins: collagen IV (C, mouse, Southern Biotech; Cat. No. 1250–04S), fibronectin (F, bovine, Sigma; Cat No. F1141), laminin (L, mouse, Life Technologies; Cat No. 23017–015), gelatin (G, bovine, Sigma; Cat No. G1393), heparan sulfate (H, mouse, Sigma; Cat No. H4777), and Matrigel (M, mouse, BD Biosciences; Cat No. 356231) (Fig. 1A). The single factor ECMs were further mixed at equal mass ratios (ie, 1:1, 1:1:1, etc.) to obtain a total of 63 unique ECM combinations, each with 6 replicates on each ECM microarray slide. The final concentration of all ECM combinations was held constant at 0.5 mg/ml. The ECMs were deposited using an OmniGrid Accent Microarrayer (Gene Machines) onto a surface-reactive glass slides (Schott H, Nexterion) for covalent protein conjugation, as described previously^18^. The resulting combinatorial ECM microarray consisted of individual microenvironments in the form of circular “islands” that were 0.3 mm in diameter and 0.3 mm apart from neighboring islands. After covalent attachment of the ECMs, the microarray slides were air dried and transferred into vacuum sealed boxes and stored in the dark at 4 °C.

### Endothelial differentiation on ECM microarray slides

ECM microarray slides were first sterilized in 1X anti-mycotic solution (Life Technologies) for 30 minutes at 37 °C, followed by 3 washes in 1XPBS. On day 0 of differentiation, the human iPSC lines (HUF5^40^ and DOX1^41^) or ESC line (H1) were dissociated into single cells using accutase (Life Technologies) and then seeded onto the ECM microarrays at a density of 2 × 10^6^ cells per slide in differentiation media consisting of α-minimum essential medium (Invitrogen), 5% fetal bovine serum (FBS, ESC-screened, Hyclone), VEGF-A (Peprotech, 50 ng/mL) and BMP4 (Peprotech, 50 ng/mL), and denoted as DMVB medium^39, 42^. The DMVB differentiation medium was further supplemented with survival factors consisting of human ESC cloning and recovery supplement (Stemgent, 1X), Rho-associated, coiled-coil containing protein kinase (ROCK) inhibitor (Calbiochem, 10 µM) and neurotrophin-3 (Sigma, 50 ng/mL). The cells were redistributed through gently rocking the ECM microarrays every 30 minutes to promote uniform cell distribution. After 6 hours of cell seeding, the unbound cells were washed away and the medium was replaced with fresh DMVB medium. The cell-seeded ECM microarrays were incubated at 37 °C with 5% CO_2_ overnight. On the following day, the medium was changed to fresh DMVB medium in the absence of survival factors. The next day, the cells on the ECM microarrays were switched to a similar differentiation medium in the absence of BMP4 (denoted as DMV medium) for an additional three days. After five days of differentiation in total, the cells on the ECM microarray slides were fixed with 4% paraformaldehyde for immunofluorescence staining.

### Endothelial phenotypic marker expression of CD31 on ECM microarrays

In brief, the cells on the ECM microarray slides were permeabilized in 0.1% Triton-X100, blocked in 1% bovine serum albumin, and incubated with antibody targeting the endothelial phenotypic marker, CD31 (Dako) for 16 hours at 4 °C. After primary antibody incubation, the samples were incubated with Alexa Fluor-488-conjugated goat anti-mouse secondary antibody (Life Technologies), followed by incubation with Hoechst 33342 nuclear dye (Life Technologies). Each ECM microarray slide was imaged by the ImageXpress Micro high-content imaging system (Molecular Device). Automated images were acquired for each individual ECM island in the channels of 488 (CD31) and Hoechst 33342 using 10X objectives at a focal plane that gave the maximum fluorescent signal for each channel.

The acquired images were analyzed using MetaXpress software (version 5.0) to measure the integrated fluorescence density of CD31 staining in each ECM island, after thresholding above the background fluorescence (n = 5 of iPSCs (HUF5); n = 3 of iPSCs (DOX1); n = 5 of ESCs (H1)). The integrated density for each ECM island was normalized to total cell nuclei based on the corresponding Hoechst 33342 image. For comparison of data between independent ECM microarray slide, the data was normalized by the Z-score method as described by Brafman *et al*.^19^ in which Z_*i*_ = (X_*i*_ − μ)/σ, where X_*i*_ is the log_2_ transformed data for ECM composition *i*, μ is the averaged log_2_ transformed data through the entire ECM slide, and σ is the standard deviation of the log_2_ transformed data among all spots on each array. Data from replicate spots (n* = *6 per ECM composition) were averaged for each microarray slide. A heat map was generated using Multiple Experiment Viewer (MeV, The Perl Foundation) by plotting the Z-scores from each ECM slide using a color code of red and blue representing higher and lower intensities, respectively, relative to the global average. For the ease of describing the ranked ECM combinations derived from heat maps, the ECM compositions were clustered into six tiers, where Tier I compounds were associated with the highest normalized protein expression of CD31.

### Validation of combinatorial ECM effects on CD31 expression using cell culture chamber slides

Additional experiments were performed using conventional cell culture settings in order to confirm the results obtained from ECM microarrays. In particular, ESCs (H1) were cultured on 4-well chamber slides that were pre-coated for 2 hours with selected ECM compositions spanning the high and low tiers of the heat map: C + H + L (CHL, Tier I), C + G + H (CGH, Tier I), C + F + H (CFH, Tier I), C + H (CH, Tier I), M (Tier V), F (Tier VI), and C (Tier VI). The cells were seeded at a density of 3 × 10^3^ cells/mm^2^ and subjected to the same differentiation protocol as described above. After 5 day differentiation, cells were fixed in 4% paraformaldehyde, followed by immunofluorescence staining against CD31, VE-cadherin (Santa Cruz Biotech), CD105 (Santa Cruz Biotech) antibodies and Hoechst 33342. Imaging was performed on a Zeiss LSM710 confocal microscope under 10X objective (n ≥ 6). Using ImageJ software, images of CD31, VE-cadherin, and CD105 staining were converted into grey scale, and then the integrated intensity was measured after thresholding above the background fluorescence. To normalize the data, total nuclei in images stained for Hoechst 33342 images were quantified by first applying thresholding to reduce the background florescence and then using the particle counting function to count total nuclei.

### Fluorescence Activated Cell Sorting (FACS)

After 5 days of differentiation on dishes coated with M or CHL, adherent ESC-derived ECs were harvested using accutase (Thermo Fisher) and then blocked using 1% bovine serum albumin (BSA, Sigma) for 15 min on ice. Afterwards, the dissociated cells were incubated with both fluorescein (FITC)-labeled CD31(eBioscience) and an allophycocyanin (APC)-labeled VE-cadherin-APC antibodies (eBioscience) for 30 minutess in 1% BSA on ice. Sytox Blue dye (Thermo Fisher) was used to label the dead cells. The cells were sorted using a LSRII flow cytometer (BD Biosciences). Gating was based on the corresponding isotype antibody controls. Data was analyzed using FlowJo software.

### LDL Uptake Assay

Endothelial differentiation in ESCs (H1) was performed on CHL-coated 4-well chamber slides. Alexa Fluor 488-conjugated acetylated low density lipoprotein (Invitrogen) was incubated with the cells in differentiation media on day 5 of differentiation for 12 hours at 37 °C. The chamber slide was then fixed in 4% paraformaldehyde, followed by immunofluorescence staining against CD31 and Hoechst 33342 as described above. Fluorescence imaging was performed on a Zeiss LSM710 confocal microscope under 10X objectives.

### Gene Expression Analysis of Endothelial Markers and Integrin Subunits

To interrogate the role of integrin subunits in modulating the process of endothelial differentiation, ESCs (H1) and iPSCs (HUF5) differentiated on representative combinatorial ECMs from Tier I (CGH and CHL) were compared to lower tier single-factors (C and M) based on gene expression of integrin subunits. Samples were lysed on days 0 and 5 of differentiation for subsequent RNA purification. RNA was isolated using GeneJET RNA purification kit according to the manufacturer’s instructions (Thermo-Fisher Scientific). RNA concentration was measured using UV-Vis Spectrophotometer (NanoDrop 2000, Thermo Scientific), and cDNA was synthesized from RNA using the SuperScript II First-Strand cDNA Synthesis kit (Thermo Fisher Scientific) and a compact thermal cycler (T100 Thermal Cycler, Bio-Rad). Quantitative PCR was performed using a real time PCR System (Applied Biosystems) with the thermal profile of 50 °C for 2 minutes, 95 °C for 10 minutes, 40 cycles of 95 °C for 15 seconds/cycle, and 60 °C for 1 minute. The Taqman primers consisted of VE-cadherin, CD31, FLK1, and integrin subunits (α1, α4, α 5, αV, α6, β1, β3, β5) (Thermo Fisher Scientific). Quantitative PCRs were performed using a 7300 Real-Time PCR system (Applied Biosystems) for 40 cycles. Data was analyzed based on the ΔΔCt method, and then normalized to housekeeping gene (GAPDH), and expressed as relative fold changes^43^.

As a basis for comparison, we also interrogated integrin subunit gene expression using a second endothelial differentiation protocol, as described previously^39^. Briefly, confluent ESCs were transferred to ultra-low adhesion dishes with DMVB differentiation medium for 4 days in order to form embryoid bodies (EBs). These EBs were then plated on 0.2% gelatin-coated dishes in DMV differentiation medium for another 10 days. Samples were lysed on days 0, 4, 8, and 14 for gene expression analysis (n = 3).

### Antibody neutralization of integrin subunits

To determine the effect of integrin β3 or α1 subunits on endothelial differentiation, ESCs (H1) underwent differentiation on ECM-modified chamber slides as described above, with the modification of cellular pretreatment with anti-integrin β3 or α1 antibodies (EMD Millipore, 10 μg/mL) for 1 hour at 4 °C at the time of cell dissociation. The negative control group was treated with isotype control antibody. The neutralization antibody or isotype control was supplemented in the media (10 μg/mL) for the entire duration of endothelial differentiation. After 5 days of differentiation, the cells were fixed and immunofluorescently staining for CD31 and Hoechst 33342. Imaging and analysis was performed using Zeiss LSM710 confocal microscope and Image J, as described above. Additional samples were assayed by quantitative PCR after 5 days of differentiation.

### Statistical analysis

All data are expressed as mean ± standard deviation. The microarray data displayed in the heat map was log_2_-transformed and standardized by Z-scores as previously described^19^. Multi-factorial Analysis of Variance (ANOVA) decomposition (main effects, 2-factor, 3-factor, 4-factor, 5-factor and 6-factor interactions) was computed using software in R with Bonferroni adjustment (63 tests) using standard factorial analysis formulae^44^. In order to balance the full set of data, a null set of baseline values (ie, no ECM) was created based on the lowest yield from any of the experiments. Although this is artificial, it is conservative because it overestimates the values in the true null set. Multiple comparisons from the heat map were assessed using the Tukey’s studentized range test at a 4.11 threshold, which is appropriate for the large number of multiple comparisons^45^. For validation of ECM microarray data in large scale, ANOVA with Bonferroni adjustment was employed. For comparison between two groups, a Student’s t-test was used. Statistical significance was accepted at P < 0.05.

### Data Availability

The datasets generated during and/or analyzed during the current study are available from the corresponding author on reasonable request.

## Electronic supplementary material

Supplementary Info
