# Supplementary material for: Combinatorial Extracellular Matrix Microenvironments for Probing Endothelial Differentiation of Human Pluripotent Stem Cells
Source: Sci Rep. 2017 Jul 26;7:6551. doi: 10.1038/s41598-017-06986-3 (PMC5529516; doi:10.1038/s41598-017-06986-3)
Supplement: Supplementary file 1 — Supplementary Info [file 41598_2017_6986_MOESM1_ESM.pdf]

Supplementary Files

Combinatorial Extracellular Matrix Microenvironments for Probing Endothelial Differentiation of Human Pluripotent Stem Cells

Luqia Hou, PhD[1,2], Joseph J. Kim, PhD[1,2], Maureen Wanjare, PhD[1,2], Bhagat Patlolla[1], John Coller, PhD[3], Vanita Natu[3], Trevor J. Hastie, PhD[4,5], Ngan F. Huang, PhD[1,2,6 #]

[1] Stanford Cardiovascular Institute, Stanford University, Stanford, CA, USA
[2] Veterans Affairs Palo Alto Health Care System, Palo Alto, CA, USA
[3] Stanford Functional Genomics Facility, Stanford, CA, USA
[4] Department of Statistics, Stanford University, Stanford, CA, USA
[5] Department of Biomedical Data Science, Stanford, CA, USA
[6] Department of Cardiothoracic Surgery, Stanford, CA, USA

[#] **Address for Correspondence:**
Ngan F. Huang, PhD
Assistant Professor
Department of Cardiothoracic Surgery
Stanford University
Address: 300 Pasteur Drive, Stanford, CA 94305-5407
Tel: (650) 849-0559
Fax: (650) 725-3846
Email: ngantina@stanford.edu

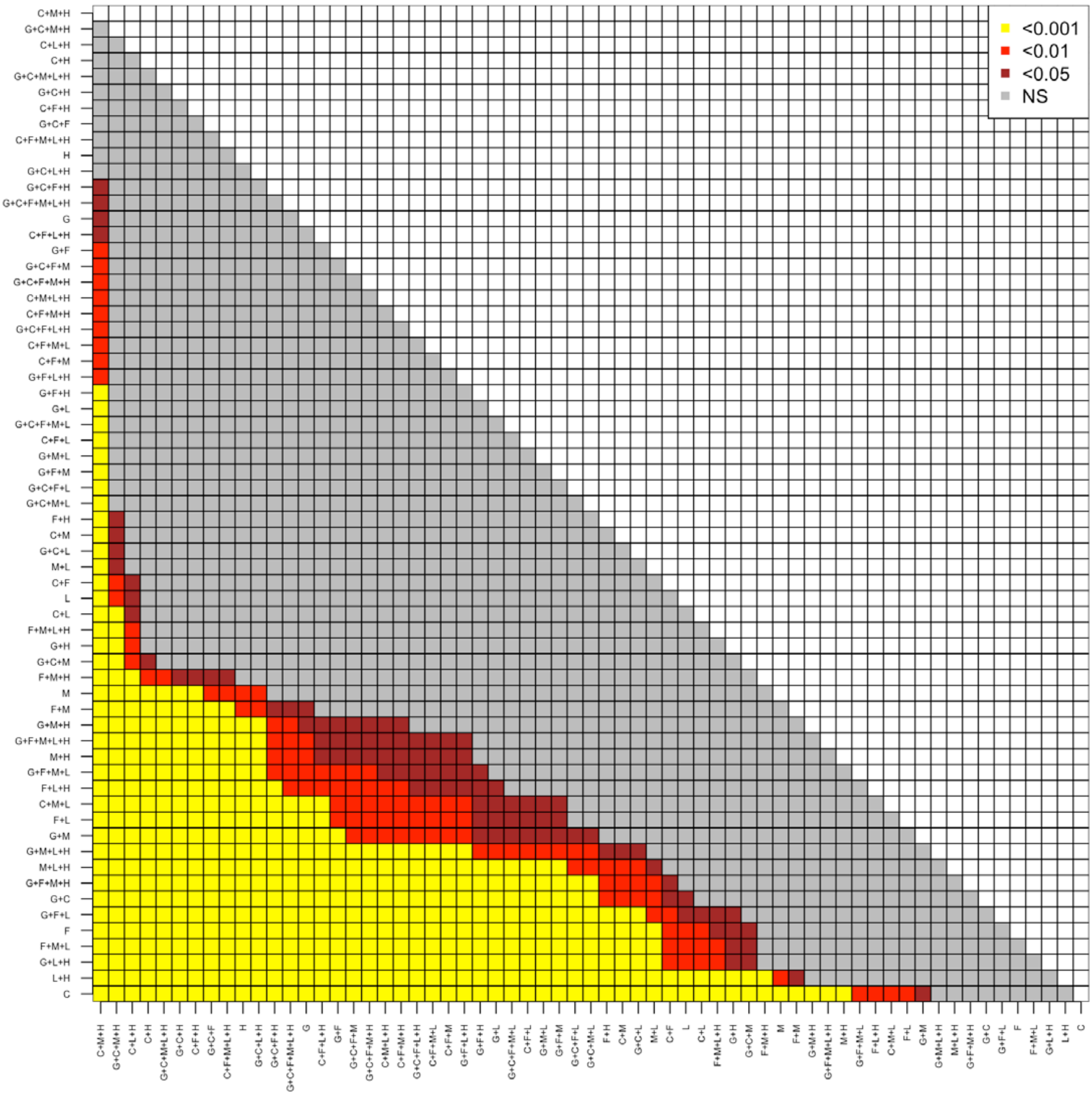

Supplementary Figure I. Multiple comparisons of endothelial differentiation on all 63 combinatorial ECMs based on CD31 expression. Normalized CD31 protein intensity was obtained using three cell lines, iPSCs (HUF5, n=5), iPSCs (DOX1, n=3) and ESCs (H1, n=5). Each multi-component or single-factor ECM was compared to each of the other 62 conditions. Multiple comparisons were assessed using the Tukey's studentized range test at a 4.11 threshold. Statistic significance between multiple comparisons was labeled in colors: cardinal: P<0.05; red: P<0.01; yellow: P<0.05.

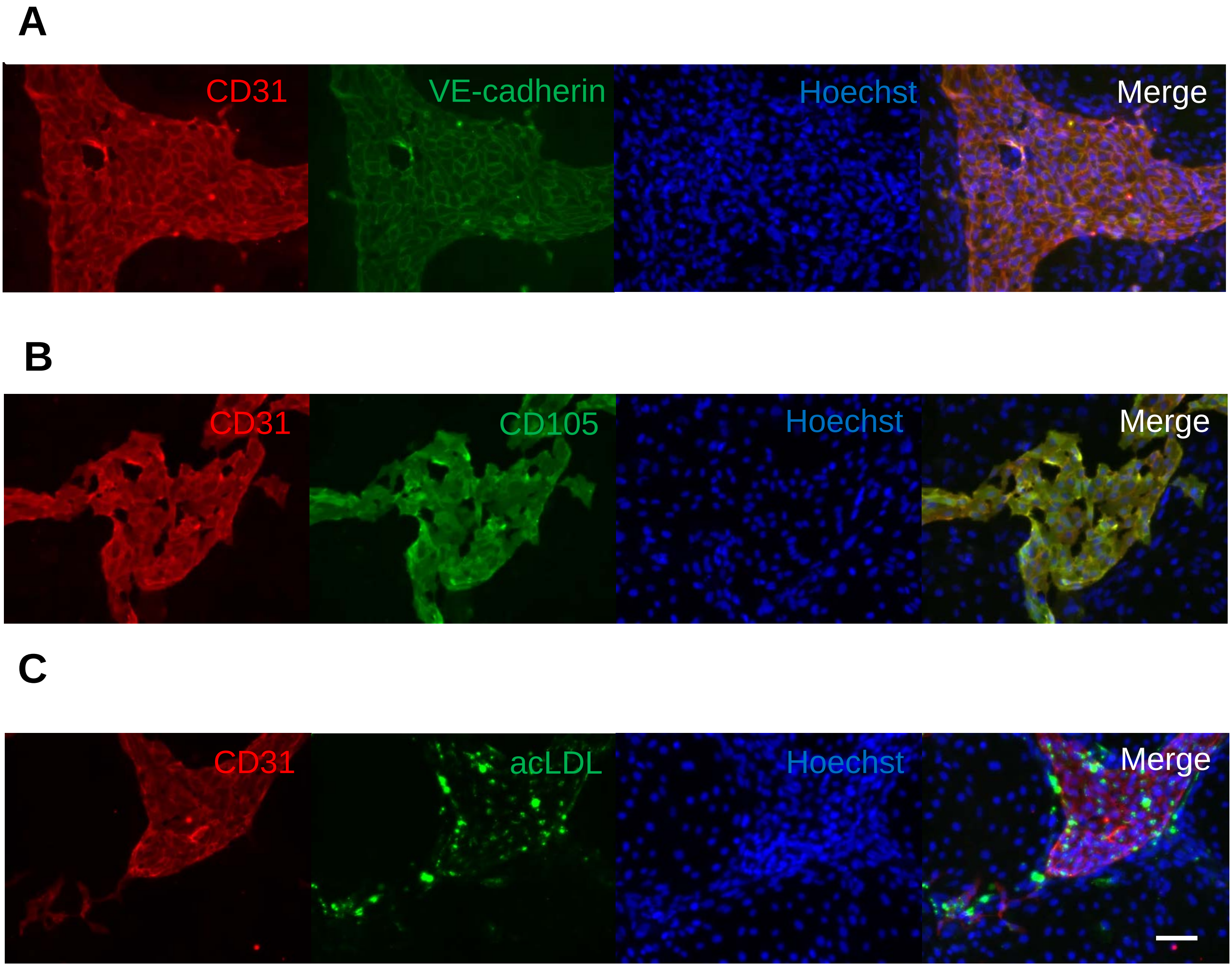

Supplementary Figure II. Characterization of endothelial differentiation of ESCs (H1) on CHL-coated chamber slides. Representative immunofluorescence staining images showed that CD31^{+} cells after 5 days of differentiation co-express (**B**) VE-cadherin and (**C**) CD105 (endoglin). Representative immunofluorescence staining images showed that CD31^{+} cells uptake acetylated low density lipoprotein (acLDL) as a function of endothelial cells. Scale bar: 100 µm.

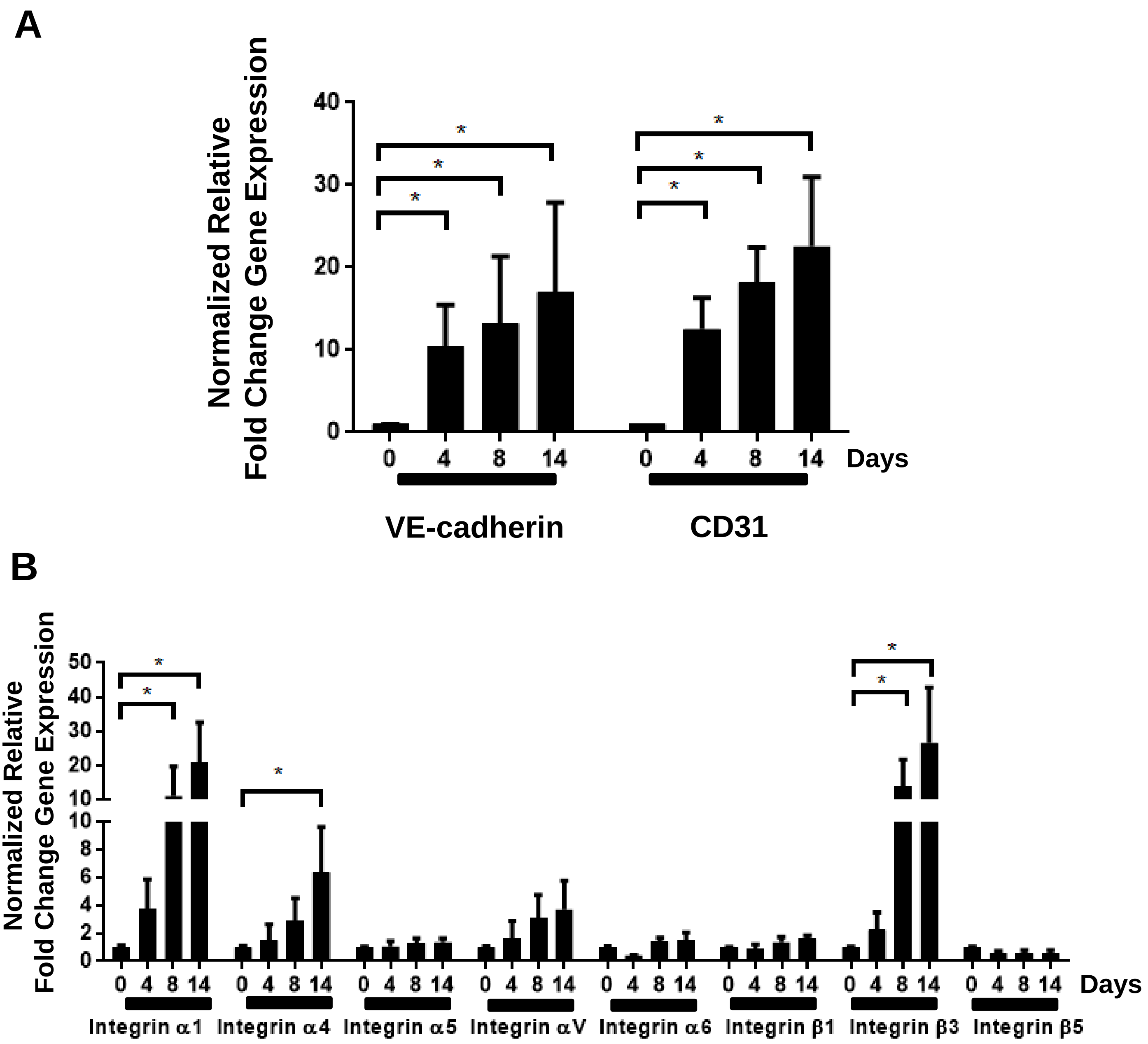

Supplementary Figure III. Integrin subunits α1 and β3 were upregulated during endothelial differentiation in human iPSCs (DOX1). (**A**) Normalized relative fold change in gene expression shows upregulation of endothelial markers VE-cadherin (>15 fold) and CD31 (>20 fold) over the course of 14 days of differentiation. (**B**) Concomitantly, integrin subunits α1, α4 and β3 were significantly upregulated over the 14 day endothelial differentiation time course (n=3). * denotes P<0.05.

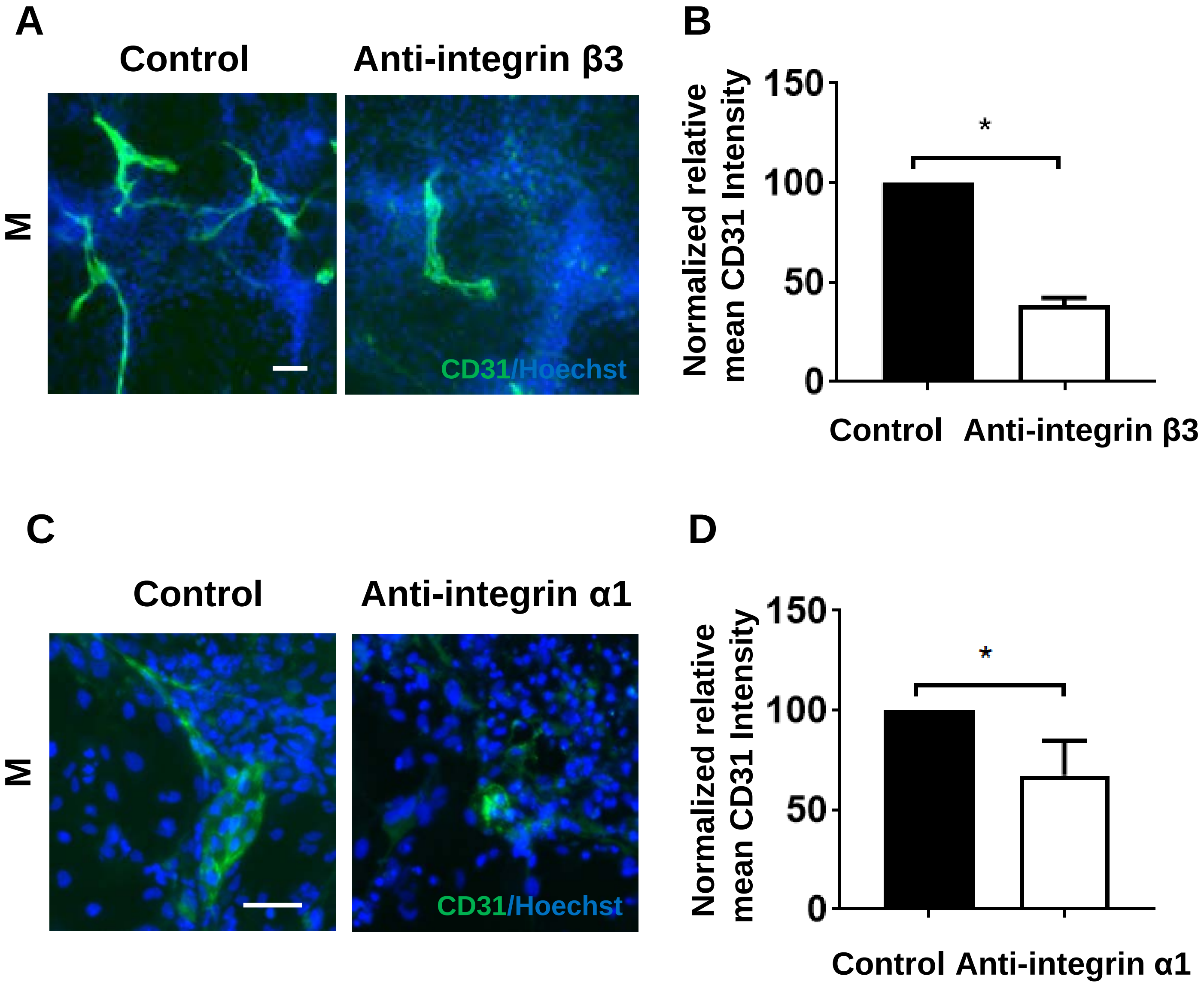

Supplementary Figure IV. Inhibition of integrin subunits β3 or α1 reduced CD31 expression in human ESCs (H1) on matrigel (M)-coated chamber slides. (**A,C**) Representative immunofluorescence staining images showed a reduction in CD31 expression when cells were treated with anti-integrin β3 (A) or anti-integrin α1 **(C)** antibody. (**B,D**) Quantification of normalized CD31 intensity showed a significant reduction (>50%) in CD31 expression in response to integrin β3 (**B**) or integrin α1 (**D**) inhibition (n=3). * denotes $P<0.05$
